# Use of Comparative Transcriptomics Combined With Physiological Analyses to Identify Key Factors Underlying Cadmium Accumulation in *Brassica juncea* L.

**DOI:** 10.3389/fgene.2021.655885

**Published:** 2021-03-29

**Authors:** Dawei Zhang, Yunyan Du, Dan He, Dinggang Zhou, Jinfeng Wu, Jiashi Peng, Lili Liu, Zhongsong Liu, Mingli Yan

**Affiliations:** ^1^School of Life Science, Hunan University of Science and Technology, Xiangtan, China; ^2^Hunan Key Laboratory of Economic Crops Genetic Improvement and Integrated Utilization, Xiangtan, China; ^3^Oilseed Research Institute, Hunan Agricultural University, Changsha, China

**Keywords:** *B. juncea* L., cadmium, comparative transcriptome analysis, physiological responses, seedling growth, transporter genes

## Abstract

The contamination of soils with cadmium (Cd) has become a serious environmental issue that needs to be addressed. Elucidating the mechanisms underlying Cd accumulation may facilitate the development of plants that accumulate both high and low amounts of Cd. In this study, a combination of phenotypic, physiological, and comparative transcriptomic analyses was performed to investigate the effects of different Cd concentrations (0, 5, 10, 30, 50 mg/kg) on *Brassica juncea* L. Our results suggest that *B. juncea* L. seedlings had a degree of tolerance to the 5 mg/kg Cd treatment, whereas higher Cd stress (10–50 mg/kg) could suppress the growth of *B. juncea* L. seedlings. The contents of soluble protein, as well as MDA (malondialdehyde), were increased, but the activities of CAT (catalase) enzymes and the contents of soluble sugar and chlorophyll were decreased, when *B. juncea* L. was under 30 and 50 mg/kg Cd treatment. Comparative transcriptomic analysis indicated that *XTH18 (xyloglucan endotransglucosylase/hydrolase enzymes), XTH22*, and *XTH23* were down-regulated, but *PME17 (pectin methylesterases)* and *PME14* were up-regulated, which might contribute to cell wall integrity maintenance. Moreover, the down-regulation of *HMA3 (heavy metal ATPase 3)* and up-regulation of *Nramp3 (natural resistance associated macrophage proteins 3)*, *HMA2 (heavy metal ATPase 2)*, and *Nramp1 (natural resistance associated macrophage proteins 1)* might also play roles in reducing Cd toxicity in roots. Taken together, the results of our study may help to elucidate the mechanisms underlying the response of *B. juncea* L. to various concentrations of Cd.

## Introduction

Cadmium (Cd) has long been recognized as a serious environmental problem due to the expansion of industrialization and urbanization (Li et al., 2014; Zhao et al., 2015). As an unessential element for plant growth and development, Cd is inadvertently taken up by roots and translocated in shoots, which may affect nearly every aspect of plant physiology and biochemistry (Uraguchi et al., 2009; Clemens et al., 2013). Excessive Cd accumulation in plants can disrupt the absorption and translocation of essential elements (Peng et al., 2017a; Rizwan et al., 2019) and dissolve the thylakoid membranes of chloroplasts (Ali et al., 2013b, 2014), resulting in the overproduction of reactive oxygen species (Ali et al., 2013a, 2015; Yan H. et al., 2016; Kapoor et al., 2019; Alam et al., 2020). Several strategies have been established to prevent the negative effects of Cd, such as immobilization, soil washing, and phytoremediation using plants (Wuana and Okieimen, 2011; Li et al., 2014). Among these strategies, plant breeding or engineering could represent an efficient approach to either increase the Cd concentration in non-edible plant organs for phytoremediation or reduce that in edible plant organs (Grant et al., 2008; Mahar et al., 2016; Tang et al., 2017). Therefore, improving understanding of the Cd uptake, transport, and accumulation mechanisms could be very helpful in developing both high and low Cd accumulation plants.

The cell wall (CW) is the first structure of plant cells to come into contact with heavy metals. When plants are under Cd stress, the cell walls act not only as protective barriers surrounding protoplasts that directly isolate Cd outside the cell but also as biosorbents, primarily by immobilizing Cd in the cell wall to reduce cellular damage (Parrotta et al., 2015; Feng et al., 2018). The primary CW is composed of components such as cellulose, hemicellulose, and pectin. Among these components, the methylesterification of pectin is controlled mainly by pectin methylesterases (PMEs), and those PMEs contribute to cell wall integrity maintenance when plants are under stress (Lionetti et al., 2017; Peng et al., 2017b; Xiao et al., 2020). Moreover, the xyloglucan endotransglucosylase/hydrolase enzymes (XTHs) are involved in hemicellulose synthesis and play important roles in responses to Cd (Xiao et al., 2020).

Moreover, several different types of transporter genes have been determined to participate in different transport processes to mediate the overall Cd accumulation in plants (Clemens et al., 2013). For root uptake, Nramp5 (natural resistance associated macrophage proteins 5) has been recognized as a major transporter in rice and barley. Knockout of *Nramp5* using RNAi or CRISPR/Cas9 and resulted in a dramatic decrease in the Cd concentration in the roots, shoots, and grains of rice (Sasaki et al., 2012; Wu et al., 2016; Tang et al., 2017). Other Nramp proteins, such as Nramp1, also play important roles in Cd uptake from soil (Takahashi et al., 2011; Tiwari et al., 2014; Zhang W. et al., 2020). After absorption by roots, HMA1 (heavy metal ATPase 1) is involved in chloroplast Cd detoxification, HMA3 is associated with high Cd accumulation in root vacuoles, and HMA2 contributes to the translocation of Cd during the xylem loading process (Takahashi et al., 2012; Yan J. et al., 2016; Zhao et al., 2019). Moreover, LCT1 (low-affinity cation transporter 1), MTL (metallothionein-like protein), and other transporters are also important components that contribute to Cd transport and accumulation (Uraguchi et al., 2014; Peng et al., 2017a; Wang et al., 2019).

*Brassica*, which belongs to Brassicaceae, includes a number of agriculturally important crops and contributes one of the major sources of edible oil (*B. napus* L. and *B. juncea* L.) and vegetables (*B. oleracea* L., *B. rapa* L., and *B. juncea* L.) worldwide (Wang et al., 2011; Liu et al., 2014; Yang et al., 2016; Sun et al., 2017). Meanwhile, some *Brassica* species also exhibit the potential to tolerate and accumulate heavy metals as hyperaccumulator plants (Mourato et al., 2015; Mahar et al., 2016). For instance, *B. juncea* L. accumulates Pb, Au, Cd, Zn, and *B. nigra* absorbs Pb. Some cultivars of *B. rapa* and *B. oleracea* are relatively tolerant of Cd and could represent good candidates for crop rotation or phytoremediation in contaminated areas (Zhou et al., 2016; Yu et al., 2017; Bernard et al., 2018; Du et al., 2020). Several studies also suggest that Cd stress could hamper seedling growth by decreasing the biomass and chlorophyll content and disrupting the redox reaction balance in *B. napus* L. and *B. juncea* L (Ali et al., 2013b, 2015; Yan H. et al., 2016; Kapoor et al., 2019; Du et al., 2020). However, the mechanisms underlying Cd accumulation in the economically important plant *B. juncea* L. have not been fully elucidated to date. Herein, a combination of phenotypic, physiological, and comparative transcriptomic analyses were performed to investigate the effects of various concentrations of Cd on plant growth, element uptake, oxidative damage (MDA accumulation), antioxidant enzymatic activities (SOD, POD, and CAT), and non-enzymatic products (chlorophyll, soluble sugar and protein). The gene expression patterns in the roots and leaves of *B. juncea* L. were subjected to long-term Cd exposure and investigated to identify the key factors involved in Cd transport and tolerance.

## Materials and Methods

### Plant Materials and Cd Treatment

The reference soil was collected from agricultural surface layer soils (0–20 cm) from a suburban area of Xiantan (27°91′05″N, 112°92′60″E, China). The physicochemical properties of these soils (pH 5.56, total nitrogen 1803.75 mg/kg, total phosphorus 921.04 mg/kg, available potassium 135.41 mg/kg, total organic carbon 1.62%, and an initial Cd concentration of 0 mg/kg) were measured by the Institute of Soil Science, Chinese Academy of Sciences (Nanjing, China), as described by Kong et al. (2018). Six weeks before the beginning of the experiments, approximately 240 kg reference soil was sieved through a 5-mm mesh and dried naturally until total dehydration. Subsequently, the reference soil was divided into five soil aliquots. One aliquot of non-contaminated reference soil was used as a control (CK), and four aliquot soils were spiked with CdCl_2_ aqueous solution to obtain four concentrations of Cd treatments: 5 mg/kg (Cd5), 10 mg/kg (Cd10), 30 mg/kg (Cd30) and 50 mg/kg (Cd50). All soil samples from each treatment were mixed again for 2 weeks to homogenize, and 8 kg of soil was subsequently added to each pot (43 cm length × 14 cm wide × 19 cm high). All treatments were arranged randomly with six replicates (pots).

The *B. juncea* L. cultivar “Purple Leaf Mustard” was used in this experiment. Seeds of oilseed rape were sown directly and 10 seedlings were maintained in each pot. The plants were irrigated with distilled water and grown under the same environment (20–25°C with natural lighting) in a greenhouse located at Hunan University of Science and Technology. Since no significant difference could be observed after short-term Cd exposure in *B. oleracea* (Bernard et al., 2018). Considering that *B. juncea* L. is widely regarded as a good plant for phytoremediation purposes (Mourato et al., 2015; Du et al., 2020). The seedlings were harvested at 50 days after sowing to evaluate their phytoremediation potential after long-term exposure to Cd. Plant height and fresh weight were calculated manually and the total leaf area was measured using Image J software. All of these phenotypic indexes were estimated in six biological replicates per treatment.

### Determination of Antioxidant Enzymes, Soluble Sugar, and Protein, Chlorophyll Content

The determination of antioxidant enzyme activities, including SOD, POD, and CAT, was performed according to the method described by Li et al. (2013) with some modifications. In brief, approximately 0.1 g fresh seedlings were ground in liquid nitrogen and subsequently suspended in 0.9 mL of 50 mM potassium phosphate buffer (pH 7.8). The homogenate was centrifuged at 10,000 g for 10 min, and the supernatants were collected for antioxidant enzyme activity measurement. Commercially available assay kits (Nanjing Jiangcheng Bioengineering Institute)^1^ were used to determine the activities of superoxide dismutase (SOD, hydroxylamine method), catalase (CAT, visible light method), and peroxidase (POD) according to the manufacturer’s instructions as well as the protocol described by Li et al. (2013). The activity of SOD, CAT, and POD was measured using a Cary 60 UV-Vis (Agilent Technologies, Palo Alto, United States) by the absorbance at 550, 405, and 420 nm, respectively.

MDA was determined according to the method outlined by Yan H. et al. (2016), with some modifications. In brief, approximately 0.3 g fresh seeding was homogenized in 3 ml of 5% (w/v) trichloroacetic acid (TCA) and centrifuged at 10,000 g for 10 min. The supernatant (1 ml) with 4 ml 0.5% (w/v) thiobarbituric acid (TBA) was heated in a water bath for 20 min at 100°C and immediately chilled on ice. Next, the homogenate was centrifuged at 4°C and 3,000 g for 10 min to collect the supernatant. Finally, the absorbance at 532 nm was measured using a Cary 60 UV-Vis (Agilent Technologies, Palo Alto, United States), and the MDA content was calculated.

The content of soluble sugar was determined by the anthrone colorimetry method (Dubois, 1956), using the assay kit provided by Nanjing Jiangcheng Bioengineering Institute. About 0.1 g of fresh seedlings were milled in liquid nitrogen and homogenized in 1 ml of distilled water. The homogenate was incubated in a bath (95°C and10 min) and then centrifugated at 6,000 g for 10 min at room temperature. Subsequently, the supernatant was collected. After that, 2 ml of anthrone reagent was added to the supernatant and the mixture was incubated in a bath (95°C and 10 min). After chilling to room temperature, the absorbance of the mixture at 620 nm was measured using a Cary 60 UV-Vis (Agilent Technologies, Palo Alto, United States).

The content soluble protein was determined by the Coomassie Brilliant Blue G250 staining method (Bradford, 1976) using the assay kit provided by Nanjing Jiangcheng Bioengineering Institute. Absorbance was recorded spectrophotometrically at 595 nm (Cary 60 UV-Vis, Agilent Technologies, Palo Alto, United States) using bovine serum albumin as a standard.

The chlorophyll content in leaves was determined by the acetone extraction method (Kim et al., 2017). In brief, about 0.1 g of fresh leaves were ground in liquid nitrogen and 5 mL of 80% acetone was added. Then, the solution was mixed gently in the dark for 24 h to protect the chlorophyll from light damage. The mixture was centrifuged at 12, 000 g for 15 min. Chlorophyll levels were measured using a Cary 60 UV-Vis (Agilent Technologies, Palo Alto, United States) by the absorbance at 663 and 645 nm.

All of these physiological indexes were estimated in six biological replicates per treatment.

### Determination of Cd Concentrations

The roots and leaves of *B. juncea* L. seedlings were harvested separately, washed with distilled water, and dried at 75°C until dehydration. All samples were ground to a fine powder, digested in 70% nitric acid at 100°C for 2 h, and diluted with ultrapure water for metal determination. The rhizosphere soils were ground to a fine powder and digested in hydrochloric acid (37%): nitric acid (70%) at a ratio of 3:1, boiled for 2 h, and diluted with 5% nitric acid after cooling. The concentrations of Cd in leaves, roots, and rhizosphere soils (four replications per treatment) were determined by flame atomic absorption spectrophotometry (Agilent 200 AA, Agilent Technology Co., Ltd.). Meanwhile, the concentrations of Cd and other elementals in the leaves were further confirmed by inductively coupled plasma-mass spectrometry, as described by Peng et al. (2017a). The translocation factor was calculated (translocation factor = Cd concentration in leaves/Cd concentration in roots) to evaluate the relative translocation of Cd from roots to shoots. The bioconcentration factor was calculated (bioconcentration factor = Cd concentration in leaves/Cd concentration in rhizosphere soils) to evaluate the relative bioconcentration ability of plants.

### Libraries Construction and Sequencing

Considering that no significant difference in phenotype was observed between Cd5 and CK, and the plants under Cd50 treatment were too small and not numerous enough for sample collection. The roots and leaves growing under CK, Cd10, and Cd30 were collected for further RNA-seq. Each treatment had three biological replicates, resulting in a total of 18 samples. Total RNA of samples was extracted using TRIzol^®^ Reagent (Invitrogen) and monitored on 1% agarose gels. Moreover, a NanoPhotometer^®^ spectrophotometer (IMPLEN, CA, United States), and RNA Nano 6000 Assay Kit of the Bioanalyzer 2100 system (Agilent Technologies, CA, United States) were also used to check the purity and integrity of RNA. Samples with RIN scores of ≥7.5 were utilized for further sequencing. Sequencing libraries were generated using the NEBNext^®^ UltraTM RNA Library Prep Kit for Illumina^®^ (NEB, United States) following the manufacturer’s recommendations. The libraries were sequenced on an Illumina NovaSeq platform and 150 bp paired-end reads were generated by Novogene company. All the sequencing data were submitted to NCBI SRA (accession number: PRJNA592031).

### Reads Mapping and Quantification of Gene Expression Levels

Clean reads were obtained by removing reads containing adapter, poly-N, and low-quality reads. Paired-end clean reads were aligned to the *B. juncea* L. reference genome using Hisat2 v2.0.5 (Yang et al., 2016). FeatureCounts v1.5.0-p3 was used to count the reads numbers mapped to each gene. Differential expression analysis was performed using DESeq2 and the expression levels of individual genes were quantified using the FPKM (FragmentsPer Kilobase per Million) method. The resulting *P*-values were adjusted using Benjamini and Hochberg’s approach for controlling the false discovery rate. Genes with adjusted *P* < 0.05 and fold changes >2 were classified as differentially expressed.

### GO Enrichment Analysis of Differentially Expressed Genes

Gene Ontology (GO) enrichment analysis of differentially expressed genes was implemented by the clusterProfiler R package (Yu et al., 2012). GO terms with corrected *P* > 0.05 were considered to be significantly enriched.

### Identification of Cd Transporter Genes in *B. juncea* L.

To identify Cd transporter genes in *B. juncea* L., 12 genes from *A. thaliana* that are already known to be involved in Cd transport were chosen. Blastp was performed in BRAD^2^ based on their sequence similarity. The Conserved Domain Database^3^ was also used to confirm that these proteins contained a specific domain for metal transport. Using E < e^–105^ as a cut-off, a total of 49 genes were identified as Cd transporter genes in *B. juncea* L. (Supplementary Table 1).

### Validation of the Expression Data by qRT-PCR

The RNA samples for RNA-seq experiments were also used for qRT-PCR. First-strand cDNA synthesis was performed using a Thermo Fisher Scientific Revert Aid First Strand cDNA Synthesis Kit. Differentially expressed genes were randomly selected and specific primers (Supplementary Table 2) were designed for qRT-PCR using NCBI Primer-BLAST^4^. The GAPDH gene was used as an internal housekeeping gene control (Chandna et al., 2012), and SYBR Premix Ex TaqII with a Bio-Rad CFX96 Real-Time Detection System was also used according to the described previously (Zhang et al., 2018; Zhang D. et al., 2020). Semi-quantitative RT-PCR was also performed and analyzed in an electrophoresis gel to verify the expression of the two Cd-induced expression genes.

### Statistical Analysis

The correlation coefficients between phenotypic indexes and Cd concentration were determined using Pearson correlation tests. A one-way variance analysis (ANOVA) was carried out and treatment means were compared using Duncan’s multiple range tests at a significance level of *P* = 0.05 for any significant differences.

## Results

### Effect of Cd Treatment on the Phenotype of *B. juncea* L.

**FIGURE 1:**
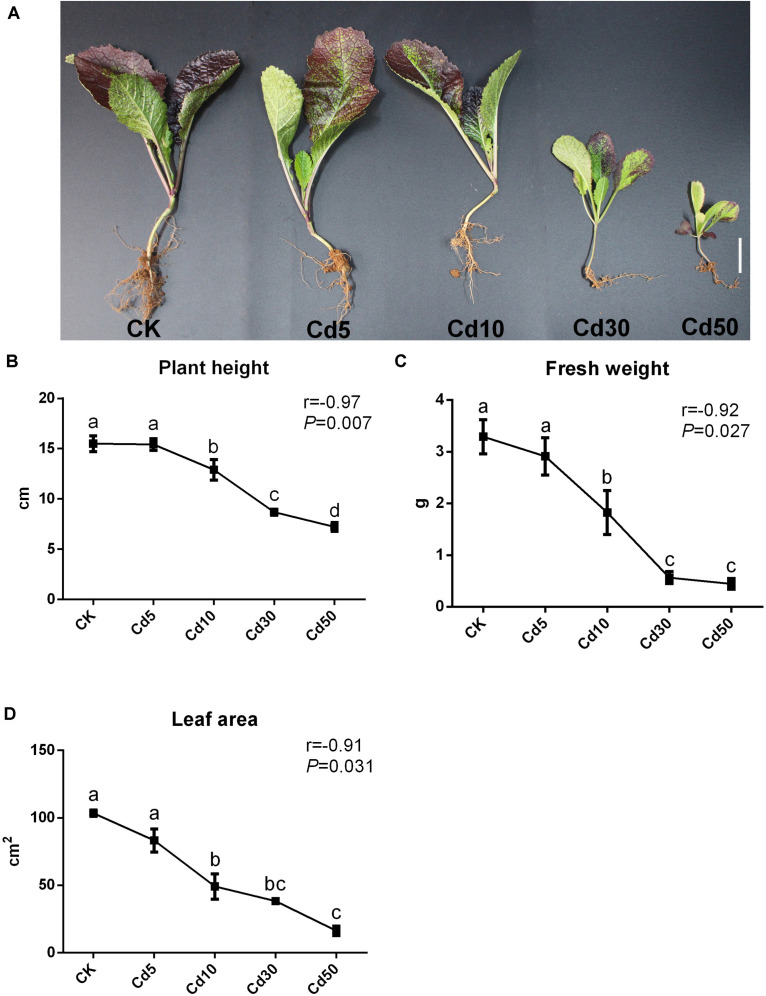
Phenotype of *B. juncea* L. under different concentrations of Cd treatment. **(A)** The phenotype of *B. juncea* L. seedlings (scale bar = 2 cm); **(B)** plant height; **(C)** fresh weight; **(D)** total leaf area; of *B. juncea* L. seedlings at 50 days after sowing. Different letters indicate significant differences at *P* < 0.05 according to Duncan’s multiple range tests.

Different concentrations of Cd treatment were observed to suppress the growth of oilseed rape seedlings to different degrees, resulting in visible effects on phenotype after 50 days of vegetative growth (Figure 1A). Although no significant difference was observed between the low concentration of Cd treatment (Cd5) and the control (CK), plant height decreased significantly with increasing Cd concentration (Figure 1B). Moreover, the total leaf area and fresh weight also decreased significantly under higher Cd concentration compared with the control (Figures 1C,D). Treatment with high concentrations of Cd (30 mg/kg and 50 mg/kg) significantly hampered seedling growth, as the plant height, total leaf area, and fresh weight decreased by approximately 44, 63.1, and 82.7% under Cd30, and 53.5, 84.5, and 86.7% under Cd50, respectively, compared to those under CK. Pearson correlation analysis demonstrated that the Cd concentration in the soils exhibited a significant negative correlation with plant height, fresh weight, and total leaf area (*r* > 0.9, *P* < 0.05; Figures 1B–D and Supplementary Table 3), suggesting that Cd treatment suppressed the growth of *B. juncea* L. seedlings in a dose-effect pattern.

### Effect of Cd Treatment on the Physiology of *B. juncea* L.

**FIGURE 2:**
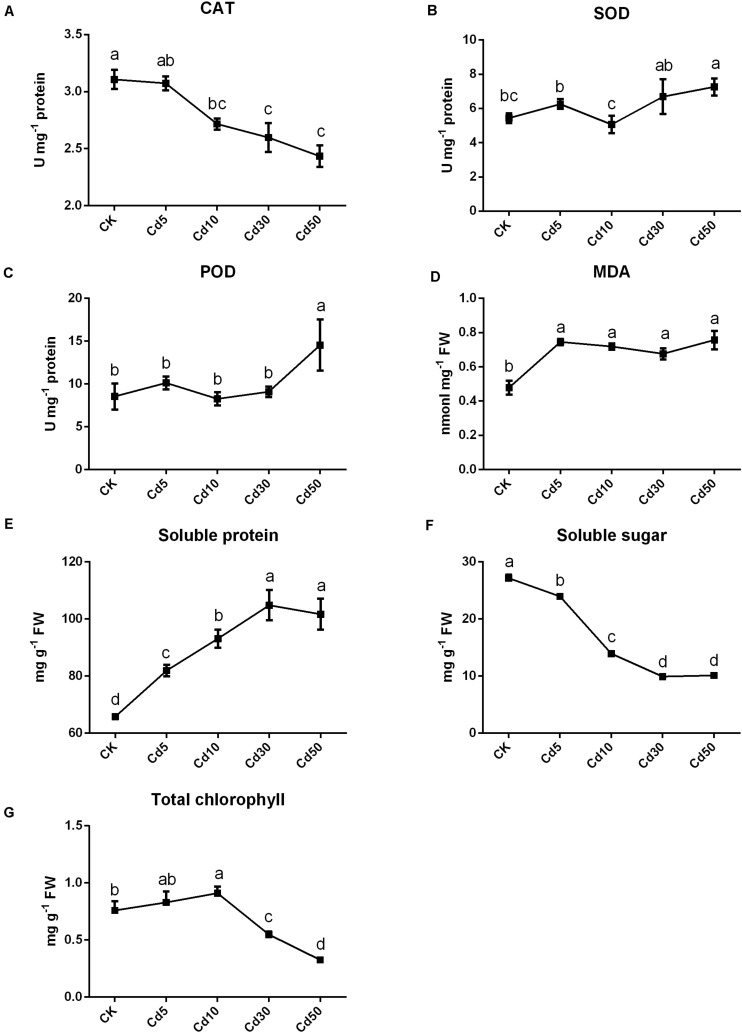
Physiological indexes of *B. juncea* L. under Cd treatment. The activities of **(A)** CAT, **(B)** SOD and **(C)** POD enzymes and contents of **(D)** MDA, **(E)** soluble sugar, **(F)** soluble protein and **(G)** total chlorophyll in *B. juncea* L. after Cd treatments. Different letters indicate significant differences at *P* < 0.05 according to Duncan’s multiple range tests.

In addition to phenotype, the physiological properties of *B. juncea* L. were also measured. The activities of CAT significantly decreased by approximately 12.5, 16.0, and 21.7% when seedlings were under the Cd10, Cd30, and Cd50 treatments, respectively, compared with CK (*P* < 0.05; Figure 2A). The activities (SOD and POD) remained stable when seedlings were under Cd5 and Cd30 treatments (*P* > 0.05) but increased significantly (about 33.6 and 70.2%; *P* < 0.05) under Cd50 compared with their CK (Figures 2B,C). Except for CK, the MDA content was increased by approximately 41.2–58.2% after Cd treatments (Figure 2D). Furthermore, the contents of soluble protein increased significantly (*P* < 0.05) in the seedling exposed to Cd compared to CK, while the contents of soluble sugar tended to decrease in *B. juncea* L. (Figures 2E,F). The chlorophyll content in leaves increased by approximately 9% under Cd5 but significantly decreased by approximately 27.8 and 57% when seedlings were under the Cd30 and Cd50 treatments, respectively, compared with CK (*P* < 0.05; Figure 2G).

### Elemental Accumulation in *B. juncea* L. Seedlings

**FIGURE 3:**
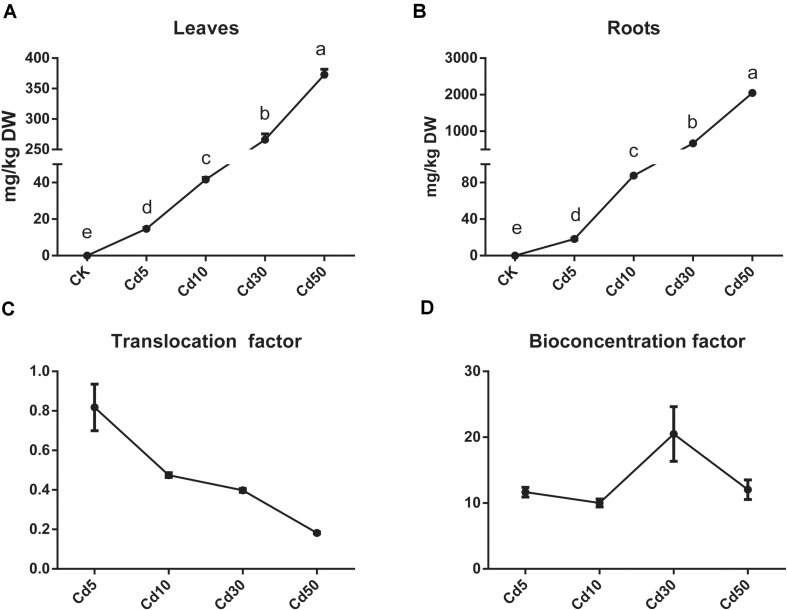
Cd accumulation in roots and leaves of *B. juncea* L. **(A)** Cd content in leaves; **(B)** Cd content in roots; **(C)** Cd translocation factor; **(D)** Cd bioconcentration factor.

The Cd content in the roots and leaves of *B. juncea* L. both increased markedly, and most absorbed the Cd accumulated in the roots, especially when the plants were exposed to high concentrations of Cd (Figures 3A,B). The Cd translocation and the bioconcentration factor varied in different Cd treatments. Plants under Cd5 (0.81) showed the highest Cd translocation efficiency, and those under Cd10/Cd30 (0.47/0.40) exhibited relatively high levels but their efficiency decreased to 0.18 under Cd50 treatment (Figure 3C). Meanwhile, the plants under Cd30 (20.5) exhibited the highest bioconcentration efficiency, because the bioconcentration factor was nearly two times that observed under Cd5(11.7), Cd10(10.0), and Cd50(12.0) treatment (Figure 3D).

Besides Cd accumulation, Cd treatment affected the accumulation of other elements in *B. juncea* L. leaves (Table 1). The change in Mg, Fe, Ca, Na, and K concentrations had the same tendency, which significantly increased by approximately 76.6, 77.5, 31.5, 894.6, and 83.4%, respectively, under Cd30. The Mn and Ca concentrations were lower under Cd10 than under CK. Meanwhile, the concentrations of Cu and Zn were not significantly different under Cd exposure.

**TABLE 1:** Effect of Cd treatment on elemental accumulation in *B. juncea* L.

	CK	Cd10	Cd30
Mg	4,777 ± 337.1^b^	4,177 ± 443.0^b^	8,435 ± 419.3^a^
Ca	20,597 ± 1,522^b^	13,020 ± 1,823^c^	27,081 ± 903.7^a^
Mn	165.0 ± 13.20^a^	67.20 ± 13.61^c^	128.6 ± 6.46^b^
Fe	120.31 ± 8.43^b^	138.25 ± 8.34^b^	213.85 ± 38.91^a^
Zn	173.3 ± 14.07^a^	143.5 ± 17.68^a^	160.2 ± 13.59^a^
Cd	–	61.25 ± 5.63^b^	274.0 ± 4.22^a^
K	66,325 ± 4,032^c^	84,437 ± 6,540^b^	110,162 ± 5,946^a^
Cu	9.36 ± 1.04^a^	10.65 ± 0.79^a^	10.75 ± 0.94^a^
Na	355.6 ± 9.02^c^	958.3 ± 53.6^b^	3531.5 ± 194.0^a^

*Elemental contents (mg/kg, dry weight) were measured in the leaves of B. juncea L.; different letters indicate significant differences at P < 0.05 according to Duncan’s multiple range tests.*

### Comparative Transcriptome Analysis of *B. juncea* L. Under Different Cd Treatments

To investigate the influence of Cd treatments on *B. juncea* L. at the gene expression level, 18 libraries (three biological replicates × 2 tissues: leaves and roots × 3 treatments: CK, Cd10, and Cd30) were constructed for high-throughput RNA sequencing (Supplementary Table 4). After removing low-quality reads containing adapters and poly-N, a total of 148.96 G clean bases were obtained, and an average of 89.1% of these were mapped to the *B. juncea* L. reference genome (Yang et al., 2016).

**FIGURE 4:**
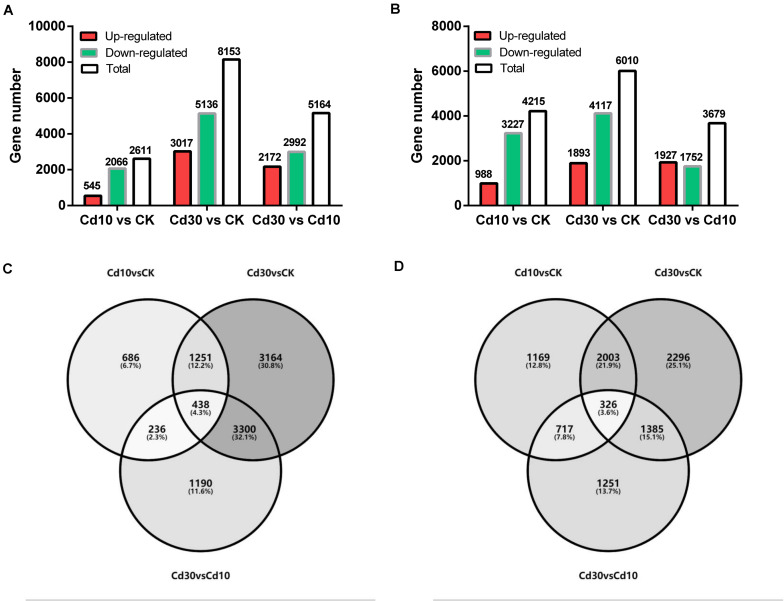
Summary of differentially expressed genes. Numbers of differentially expressed genes in leaves **(A)** and roots **(B)**. Venn diagrams of differentially expressed genes in leaves **(C)** and roots **(D)**.

Differentially expressed genes were then identified between the Cd treatment and the control (Cd10 vs. CK; Cd30 vs. CK), as well as between the Cd30 and Cd10 treatments (Cd30 vs. Cd10, Figure 4). In total, 2,611 and 4,615 genes exhibited different levels of expression between Cd10 and CK, while the number reached 8,152 and 6,010 in leaves and roots after Cd30 treatment, respectively (Figures 4A,B). More down-regulated genes (2,066, 5,136, 3,227, and 4,117) were found than up-regulated genes (545, 3,017, 988, and 1,893) in both leaves and roots when plants were under Cd treatment (*P* < 0.05, paired *t*-test). Among these differentially expressed genes in leaves, 1,689 (1,251 + 438) were shared between the two Cd treatment comparisons with CK, and 438 of them were sensitive to Cd concentration since they were also differentially expressed between the Cd30 and Cd10 treatments (Figure 4C). In roots, 2,329 (2,003 + 326) genes were common Cd responsive genes, and 326 of them were concentration-sensitive (Figure 4D).

### Functional Annotation of Differentially Expressed Genes

To identify the major functional categories of differentially expressed genes, GO enrichment analysis was conducted using clusterProfiler software (Yu et al., 2012). The genes involved in photosynthesis were significantly enriched in the leaves under Cd30 compared with CK, and the majority (98.5%, 66/67) of these genes were down-regulated (Supplementary Table 5). Moreover, GO terms associated with the cell wall, oxidoreductase activity, and response to oxidative stress were significantly enriched in the roots under Cd10 and Cd 30 compared with CK (Supplementary Table 5).

**FIGURE 5:**
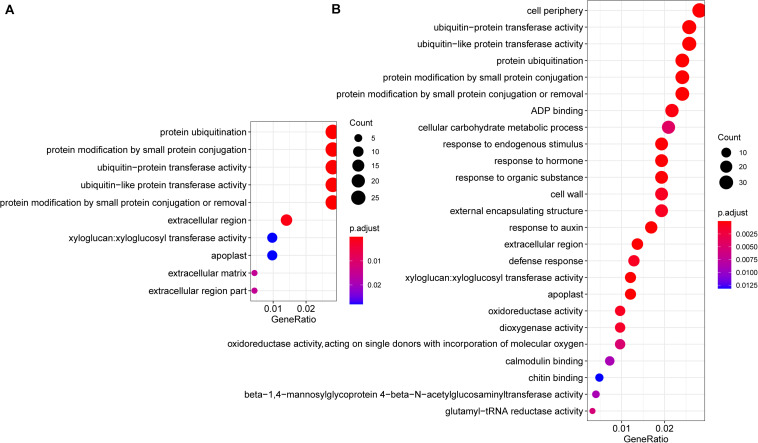
GO enrichment analysis of differentially expressed genes. The enriched GO terms of common Cd responsive genes that are differentially expressed in both Cd10 vs. CK and Cd30 vs. CK in leaves (**A**, 1,689 genes) and roots (**B**, 2,392 genes). The x-axis indicates the ratio of differentially expressed genes/background gene numbers in each GO term. The size of the circle represents the number of differentially expressed genes, while the color of the circle indicates the adjusted *P*-value enriched in each GO term.

**FIGURE 6:**
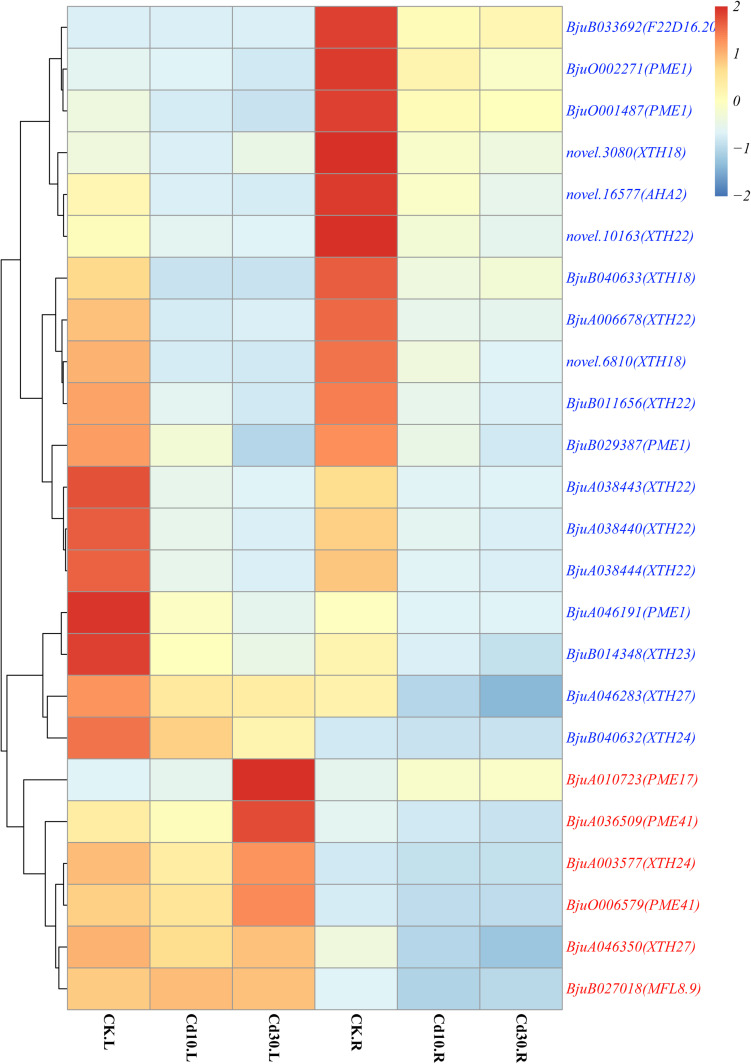
Heatmap of expressed genes involved in cell walls. Genes that were up-regulated in leaves or roots under Cd treatment are marked in red, while down-regulated genes are marked in blue. The color bar indicates the Log_2_ (average FPKM value of three replicates) in each gene.

Common Cd-responsive genes (1,689 in leaves and 2,329 in roots) were significantly enriched in protein ubiquitination, including ubiquitin-protein transferase activity, ubiquitin-like protein transferase activity, protein modification by small protein conjugation or removal, and extracellular regions, including extracellular matrix, apoplast, xyloglucan: xyloglucosyl transferase activity, and cell wall in both leaves and roots (Figure 5). Among the genes related to protein ubiquitination, the majority (93.5%, 29/31) were down-regulated under Cd treatment (Supplementary Figure 5). Regarding genes related to extracellular a, most of them (84.2%, 32/38) were also down-regulated (Supplementary Figure 3). Since cell walls play an important role in Cd responses, genes that belong to cell wall terms were also annotated and investigated. Among these 24 expressed genes, all the *PME1*(4/4), *XTH18*(3/3), *XTH22*(6/6), *XTH23*(1/1), as well as half copies of *XTH24*(1/2) and *XTH27*(1/2), were down-regulated, while *PME17*(1/1) and *PME41*(2/2) were up-regulated, after Cd treatment (Figure 6).

Genes related to the stress response, including defense response, response to endogenous stimulus, response to hormones, response to an organic substance, and response to auxin were significantly enriched in roots (Figure 5B), and 75% (18/24) of these genes were up-regulated after Cd treatment (Supplementary Figure 4). We also observed that genes related to peroxidase activity, response to oxidative stress, antioxidant activity were significantly enriched in roots after Cd30 treatment (Supplementary Table 5). Among these 46 genes related to peroxidase (POD) activity, 23 were up-regulated, and 23 were down-regulated, compared with CK (Supplementary Table 6). A similar number of up- and down- regulated POD activity genes might result in no significant difference in POD activity, as described above (Figure 2C). Although genes related to catalase (CAT) activity were not significantly enriched, about 13 genes were found to be differentially expressed under Cd treatment. Except for one gene, all the other 12 genes which encoded CAT were down-regulated either in roots or leaves under Cd10 or Cd30, which was in line with the decreasing of CAT activity above (Figure 2A).

Cd-sensitive genes (438 in leaves and 326 in roots) were also significantly enriched in protein ubiquitination, suggesting that the expression of protein ubiquitination genes was concentration-dependent (Supplementary Figure 5).

### Expression Patterns of Cd Transporter Genes

**FIGURE 7:**
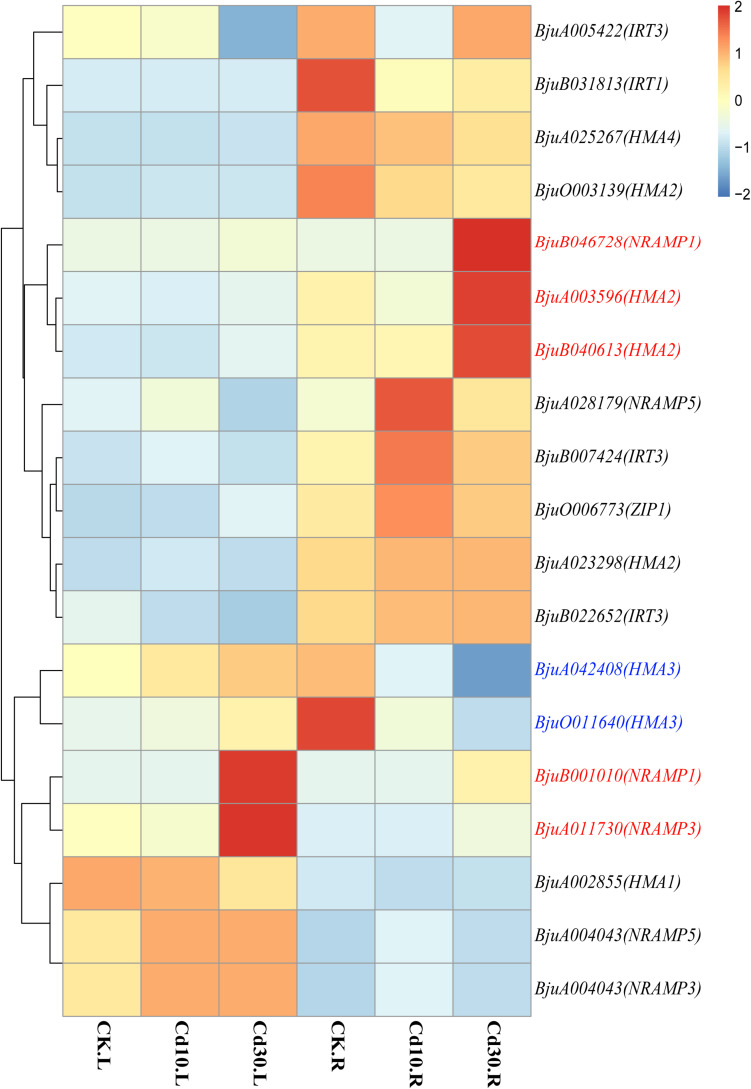
Heatmap of expressed genes encoding heavy metal transporters. Genes that were up-regulated whether in leaves or roots under Cd treatment are marked in red, while down-regulated genes are marked in blue. The color bar indicates the Log_2_ (average FPKM value of three replicates) in each gene.

**FIGURE 8:**
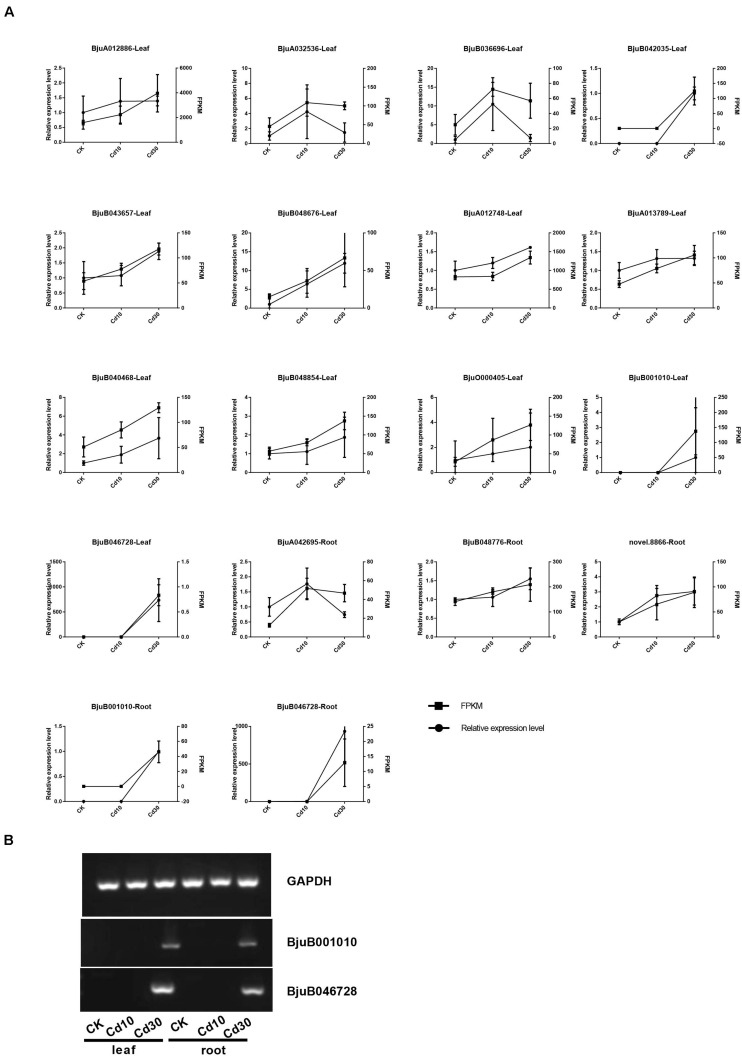
Validation of the expression data by qRT-PCR. The average FPKM value and relative expression level are shown **(A)**, and two novel genes expressed under Cd30 treatment were further confirmed by semi-quantitative RT-PCR **(B)**.

As transporters play an important role in Cd uptake, transport, and accumulation in plants, the expression patterns of 19 expressed genes (average FPKM > 1) encoding Cd transporters were also examined in *B. juncea* L. (Supplementary Table 1 and Figure 7). Genes encoding HMA3 (BjuO011640, BjuA042408) were down-regulated in roots. While the genes encoding Nramp1(BjuB001010, BjuB046728), Nramp3(BjuA011730), and HMA2 (BjuA003596, BjuB040613) were significantly up-regulated in roots, and four of them (BjuB001010, BjuB046728, BjuA011730, BjuB040613) also showed higher expression levels in leaves when plants were under Cd30 treatment. Notably, two genes, BjuB001010 and BjuB046728, which encode Nramp1, were silenced (average FPKM < 1) in both leaves and roots under the CK and Cd10 treatments but were activated (average FPKM > 10) under the Cd30 treatment. The expression of these two genes (BjuB001010 and BjuB046728) was further confirmed by quantitative and semi-quantitative RT-PCR, indicating that the expression of *Nramp1* was induced when plants were exposed to high concentrations of Cd (Supplementary Table 2 and Figure 8).

## Discussion

As an economically important plant species worldwide, *B. juncea* L. shows great potential as a rotation crop for phytoremediation in Cd-contaminated fields (Mahar et al., 2016; Yang et al., 2016). However, progress in improving the phytoremediation ability of this species remains slow since the mechanisms underlying the Cd response have not been fully elucidated in *B. juncea* L.

Several *Brassica* species have been evaluated as potential phytoextraction plants, and some of these plants could accumulate relatively high amounts of toxic metals without visible symptoms (Mourato et al., 2015). For example, no significant difference in life traits was observed in *B. oleracea* after 56 days in 5 mg/kg Cd-spiked field soil (Bernard et al., 2018). In keeping with the findings described above, we also observed no significant difference in phenotype or antioxidant enzyme activity between the *B. juncea* L. seedlings under 5 mg/kg and the control after 50 days (Figures 1, 2), indicating that the *B. juncea* L. seedlings might have a certain degree of tolerance to the relatively low concentration (≤5 mg/kg) of Cd treatment.

However, a higher concentration of Cd treatment (≥10 mg/kg, 50 days exposure) suppressed the growth of plants (Figure 1), which was consistent with the findings of Du et al. (2020) in *B. juncea* L. (≥6.65 mg/kg, 60 days exposure). The growth inhibition response to high-dose Cd exposure could be ascribed to the overproduction of reactive oxygen species (Ali et al., 2013a, 2015; Yan H. et al., 2016), reduction of photosynthetic carbon assimilation (Ali et al., 2013b, 2014), and imbalance of nutrient uptake (Ali et al., 2014; Peng et al., 2017a). Our results demonstrated that the activities of POD, SOD enzymes, contents of soluble protein, and MDA were increased in response to Cd50 treatment (Figure 2). Reactive oxygen species increased when plants were under high Cd stress, which caused MDA accumulation and oxidative stress (Yan H. et al., 2016; Du et al., 2020). Thus, increasing antioxidant enzyme activities (POD and SOD) could be beneficial for plants to remove excess reactive oxygen species (Figures 2B,C; Yan H. et al., 2016). The CAT enzyme activity, as well as the expression of related genes, showed a decreasing trend with increasing Cd concentration, suggesting that the generated reactive oxygen species might overwhelm the defense ability of the CAT enzyme (Figure 2A and Supplementary Table 6). Meanwhile, chloroplast has also been considered highly vulnerable to oxidative stress, but Cd stress could dissolve the thylakoid membranes of chloroplasts, simultaneously damaging the enzyme activity for chlorophyll photosynthesis and thereby hindering chlorophyll synthesis (Ali et al., 2014; Du et al., 2020). In our study, the chlorophyll content was significantly (27.8%) decreased (Figure 2G), and the majority (98.5%, 66/67) of photosynthesis-related genes were also down-regulated in the leaves under Cd30 (Supplementary Table 5) at the same time, indicating that photosynthesis was severely affected at both transcriptomic and physiological levels. Moreover, our study also determined that excessive Cd accumulation could affect the uptake of certain nutrients in the plants by, for instance, increasing the Na and K but decreasing Mn uptake in leaves (Table 1). An imbalance in nutrient uptake might also depress the growth of plants (Ali et al., 2014).

As the first barrier to toxic metals in the environment, CWs play crucial roles in the heavy metal response, and the synthesis and composition of CWs could also be affected at the same time (Peng et al., 2017b; Feng et al., 2018). *XTHs* were generally known to be involved in hemicellulose synthesis in primary CWs of plants and can play important roles in plant responses to Al and Cd (Zhu et al., 2012, 2013; Xiao et al., 2020). In this study, we found that most of *XTHs* (12/14), including all the copies of three *XTH18*, *XTH22*, and *XTH23* were down-regulated in *B. juncea* L. roots under Cd treatment (Figure 6). In *A. thaliana*, the Cd-tolerant ecotype had a lower expression of *AtXTHs* but a higher content of hemicellulose than the sensitive ecotype. The tolerant ecotype might sequester Cd in the CW by increasing the hemicellulose content through *XTHs* under Cd treatment (Xiao et al., 2020). Moreover, *xth31* and *xth15* mutants also exhibited increasing Al resistance and binding capacity in *A. thaliana* (Zhu et al., 2012, 2013). In accord with the above studies, the down-regulation of most *XTHs* (12/14) might also increase the hemicellulose content and Cd binding capacity in the CW of *B. juncea* L., which helped plants alleviate Cd toxicity. Regarding the *PMEs* involved in pectin polysaccharide modification, our results showed that *PME17* and *PME41* were significantly up-regulated, while *PME1* was down-regulated under Cd treatment in *B. juncea* L. A similar trend was also observed in *Arabidopsis* during pathogen infection, and the rapid and efficient activation of *AtPME17*, *AtPME20, AtPME21*, and, in particular, *AtPME41*, along with steady down-regulation of *AtPME1* could lock the decrease in pectin methylesterification and protect cell walls from further enzymatic degradation (Lionetti et al., 2017), measuring the hemicellulose and pectin contents. Therefore, we hypothesized that *XTHs* and *PMEs* might be involved in Cd tolerance in *B. juncea* L. However, further testing, such as measuring the contents of hemicellulose and pectin, as well as Cd contents in CW is still needed to confirm this hypothesis.

Cd and other metal cations can enter root cells through transporters and cation channels that are located at the plasma membrane and tonoplasts (Clemens et al., 2013). Among those transporters that participate in the process of Cd allocation and accumulation, HMA2 and Nramp1 are located in the plasma membrane and play roles in Cd loading to the xylem and root-to-shoot translocation (Wong and Cobbett, 2009; Takahashi et al., 2011, 2012; Tiwari et al., 2014; Zhang W. et al., 2020). Our study showed that a high concentration of Cd could induce the up-regulation of *HMA2* and novel expression of *Nramp1*, which might promote Cd translocation from roots to leaves in *B. juncea* L. (Figure 7). Meanwhile, HMA3 and Nramp3 were both located in the tonoplast and HMA3 was associated with high Cd accumulation in root vacuoles (Morel et al., 2009). In rice, a loss of function allele of *OsHMA3* led to increased Cd translocation from roots to shoots, whereas overexpression of *OsHMA3* produced the opposite effect (Sasaki et al., 2014; Yan J. et al., 2016). However, NRAMP3 proteins could be up-regulated upon Cd or oxidative stress, resulting in the release of metals from the vacuole (Molins et al., 2013). In our study, *HMA3* and *Nramp3* showed different expression patterns; *Nramp3* was up-regulated, but *HMA3* was down-regulated when *B. juncea* L. was under Cd30 treatment (Figure 7). In some hyperaccumulating plants or higher Cd accumulation species, the overexpression of *HMA2, HMA3, HMA4, Nramp1, Nramp3*, and other transporter genes was also associated with the enhancement of Cd accumulation and tolerance (Oomen et al., 2009; Peng et al., 2017a; Yu et al., 2017). In keeping with previous findings, the up-regulation of *HMA2*, *Nramp1* and *Nramp3* might also play a crucial role in Cd tolerance in *B. juncea* L. As to *HMA3*, we surmised that the vacuolar storage capacity of Cd in root cells might be saturated after high concentration treatment (30 mg/kg) for a relatively long time (50 days) in soils. Thus, the release of Cd from vacuolar storage by down-regulation of *HMA3* and up-regulation of *Nramp3*, along with the promotion of root-to-shoot translocation by up-regulation of *HMA2* and *Nramp1*, might reduce its toxicity in roots and enhance the bioconcentration efficiency in leaves (Figures 3D, 7).

## Conclusion

**FIGURE 9:**
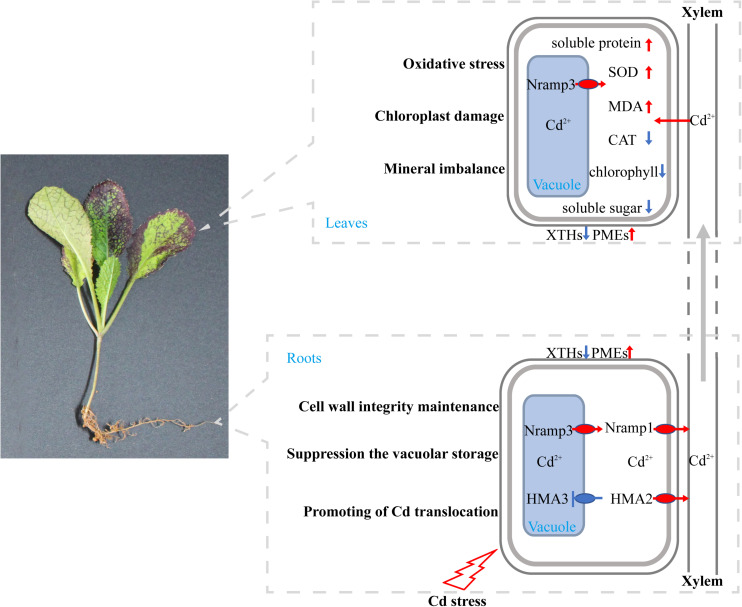
Putative model of physiological response and gene expression changes in *B. juncea* L. Genes involved in Cd transport and physiological indexes are shown. Red arrows indicate the genes or physiological indexes that were significantly up-regulated, blue arrows and blue blunt ends indicate that these genes were significantly down-regulated in the Cd treatment compared with the control. Their putative effects on *B. juncea* L. are also indicated.

Based on our results and the findings of previous studies, a putative model of physiological response and gene expression changes in *B. juncea* L. under Cd stress was proposed (Figure 9). Our results suggest that a high concentration of Cd treatment could result in oxidative stress, chloroplast damage, and mineral imbalance, thereby suppressing the growth of *B. juncea* L. seedlings. To survive under Cd stress conditions, in roots, *XTH18, XTH22*, and *XTH23* were down-regulated, but *PME17* and *PME14* were up-regulated, which might contribute to cell wall integrity maintenance and Cd toxicity tolerance. Moreover, suppression of vacuolar storage by down-regulation of *HMA3* and up-regulation of *Nramp3*, along with the promotion of root-to-shoot translocation by up-regulation of *HMA2* and *Nramp1*, might also reduce Cd toxicity in roots and enhance the bioconcentration efficiency in leaves.

## Data Availability Statement

The datasets generated for this study can be found in the online repositories. The names of the repository/repositories and accession number(s) can be found in the article/Supplementary Material.

## Author Contributions

MY and ZL conceived the study. DZ, DH, YD, DZ, and JW prepared the plant materials and samples for RNA-seq. DZ, JP, and LL analyzed the data and wrote the manuscript. All authors have read and approved the final manuscript.

## Conflict of Interest

The authors declare that the research was conducted in the absence of any commercial or financial relationships that could be construed as a potential conflict of interest.

## Footnotes

1 http://www.njjcbio.com/

2 http://brassicadb.org/brad/

3 https://www.ncbi.nlm.nih.gov/cdd

4 https://www.ncbi.nlm.nih.gov/tools/primer-blast/

## Supplementary Material

Supplementary Figure 1 Correlation coefficients between gene expression data sets among sequencing samples. R and L represent roots and leaves of *B. juncea* L. and three biological replicates are indicated as 1–3.

Supplementary Figure 2 Heatmap of differentially expressed genes involved in protein ubiquitination.

Supplementary Figure 3 Heatmap of differentially expressed genes involved in the extracellular region.

Supplementary Figure 4 Heatmap of differentially expressed genes involved in the stress response.

Supplementary Figure 5 GO categories of differentially expressed genes in all three comparisons. **(A)** Four hundred thirty-eight genes which are differentially expressed in leaves of both Cd10 vs. CK, Cd30 vs. CK, and Cd30 vs. Cd10. **(B)** Three hundred twenty-six genes which are differentially expressed in roots of both Cd10 vs. CK, Cd30 vs. CK, and Cd30 vs. Cd10. The x-axis indicates the ratio of genes vs. background gene numbers in each GO term.

Supplementary Table 1 Identification of Cd transporter genes in *B. juncea* L.

Supplementary Table 2 Corresponding primers used for qRT-PCR.

Supplementary Table 3 Pearson correlation analysis between Cd concentration and plant growth index.

Supplementary Table 4 Summary of reads filtering and mapping in 18 libraries.

Supplementary Table 5 GO enrichment of differentially expressed genes in leaves and roots under Cd treatment.

Supplementary Table 6 The different expressed genes related to peroxidase and catalase in under Cd treatment.
