# Supplementary figures and images for: Use of Comparative Transcriptomics Combined With Physiological Analyses to Identify Key Factors Underlying Cadmium Accumulation in Brassica juncea L
Source: Front Genet. 2021 Mar 29;12:655885. doi: 10.3389/fgene.2021.655885 (PMC8039530; doi:10.3389/fgene.2021.655885)

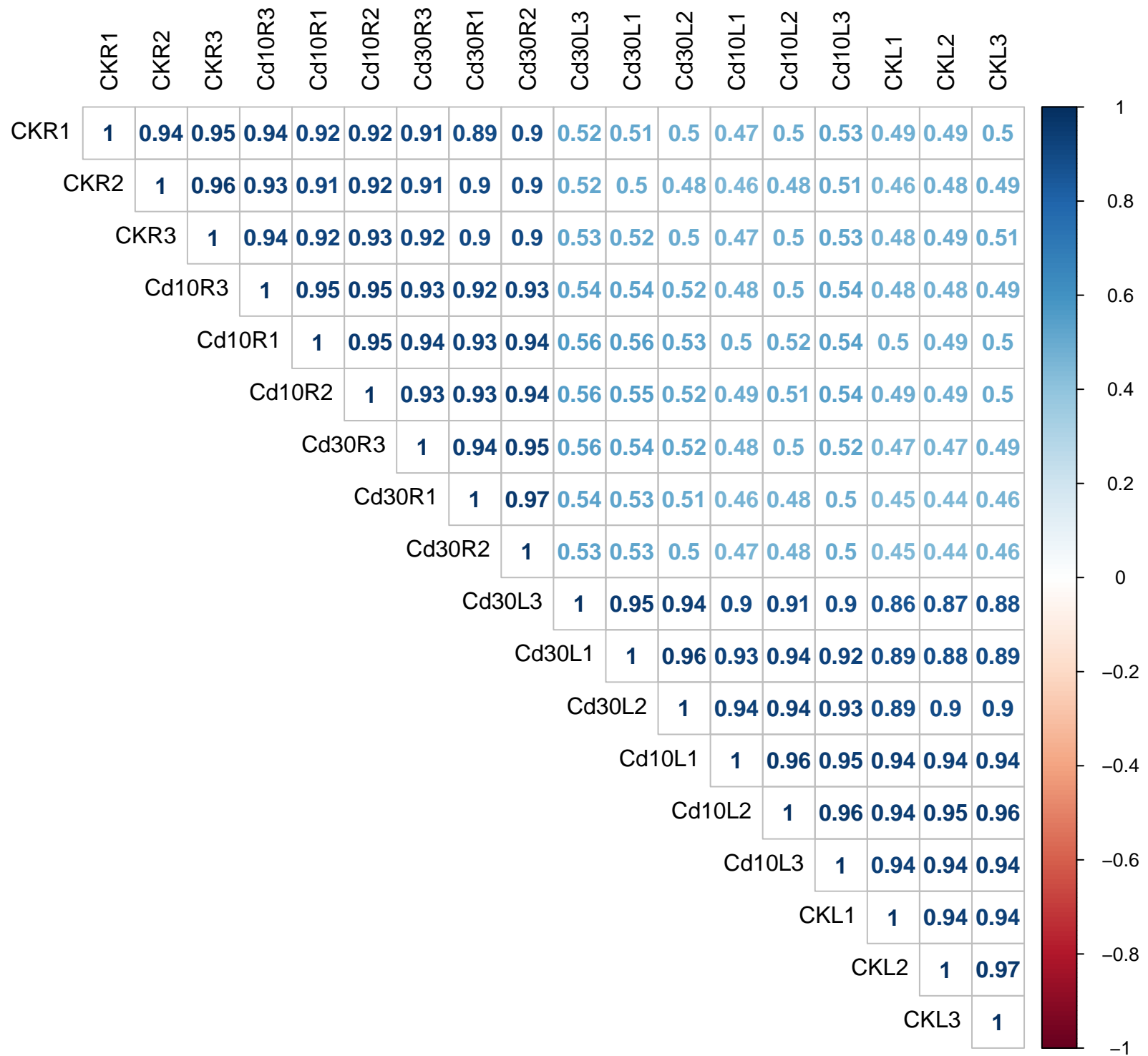

Supplement: Supplementary Figure 1 — Correlation coefficients between gene expression data sets among sequencing samples. R and L represent roots and leaves of B. juncea L. and three biological replicates are indicated as 1–3. [file Image_1.PDF]

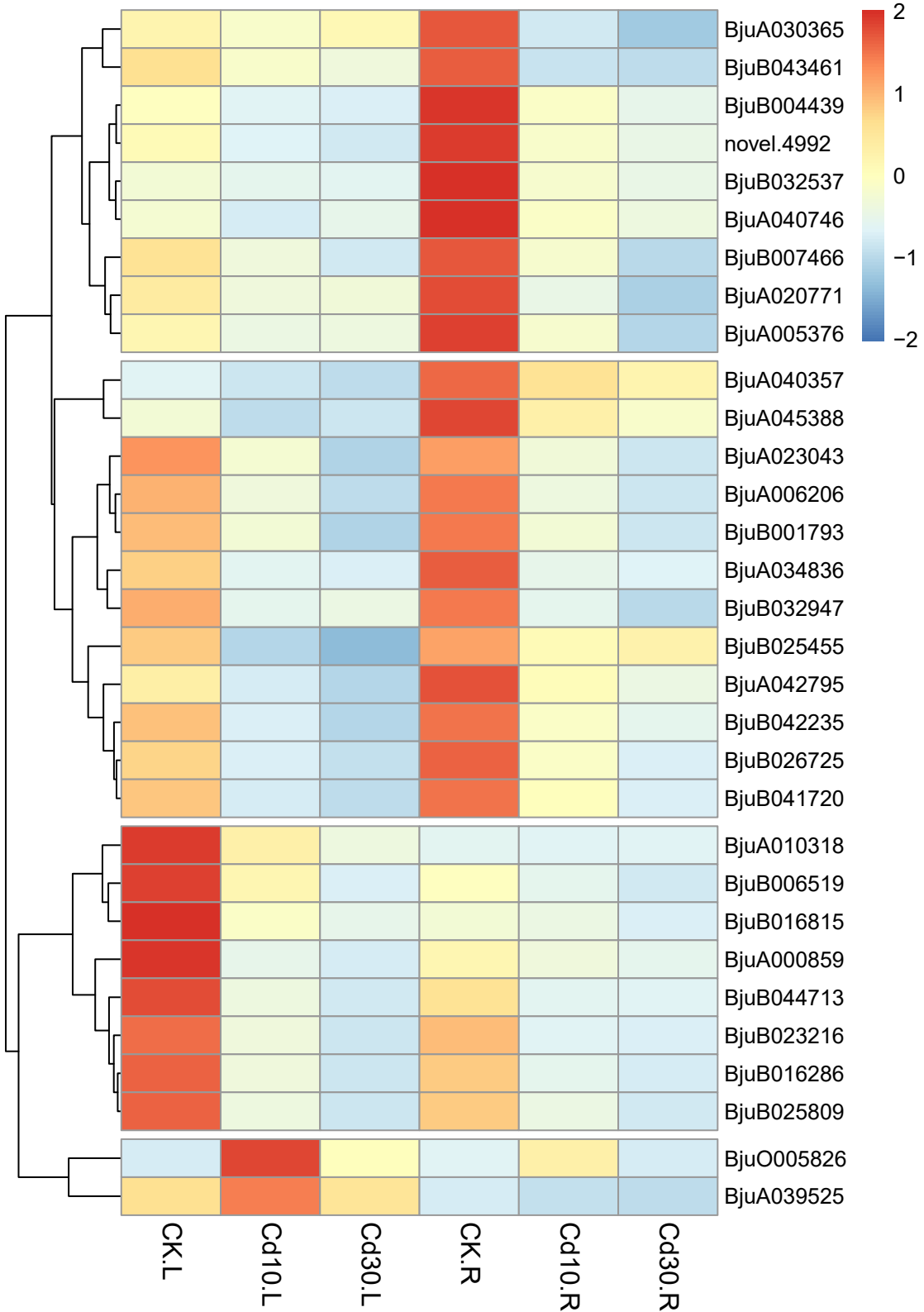

Supplement: Supplementary Figure 2 — Heatmap of differentially expressed genes involved in protein ubiquitination. [file Image_2.PDF]

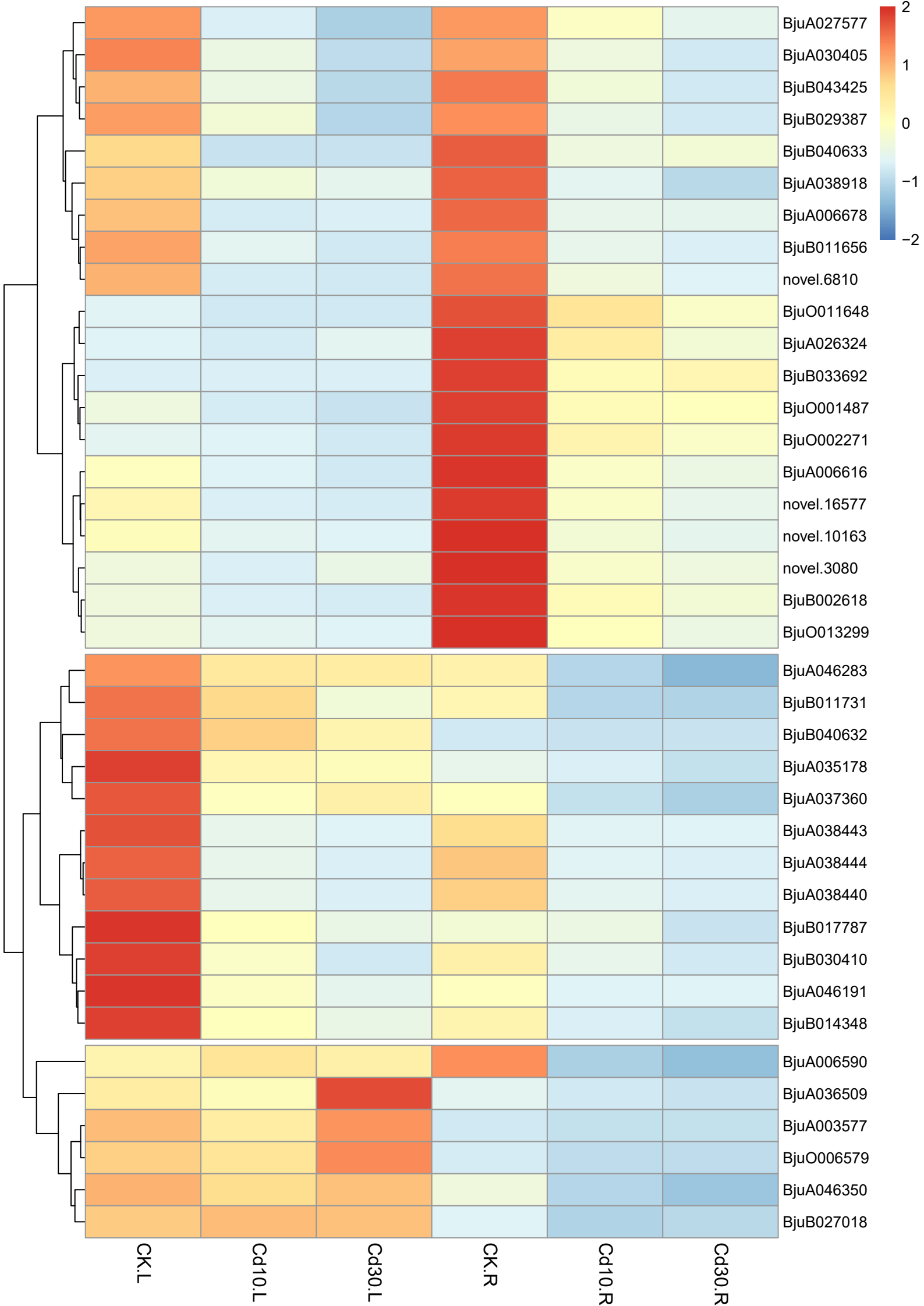

Supplement: Supplementary Figure 3 — Heatmap of differentially expressed genes involved in the extracellular region. [file Image_3.PDF]

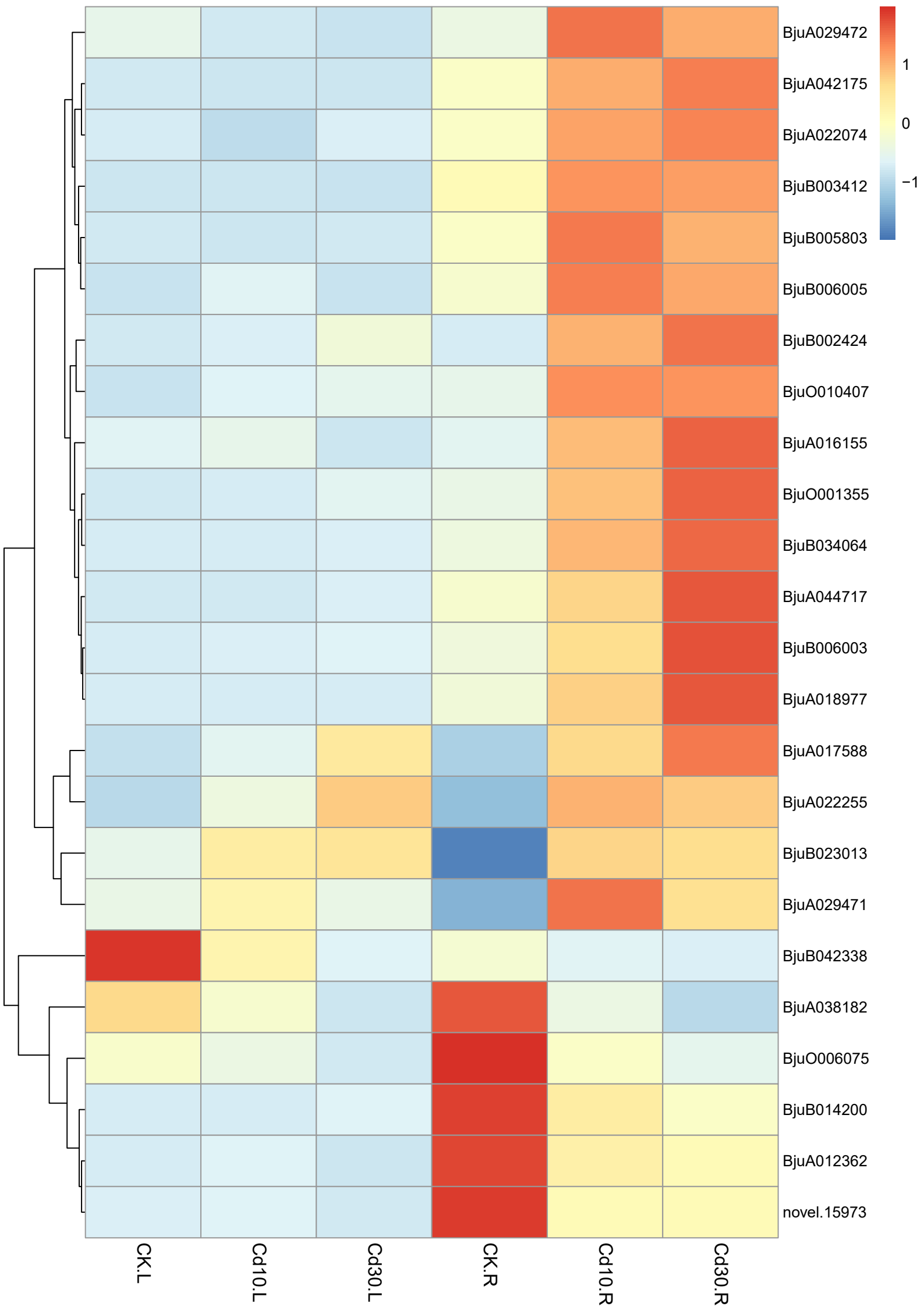

Supplement: Supplementary Figure 4 — Heatmap of differentially expressed genes involved in the stress response. [file Image_4.PDF]

A

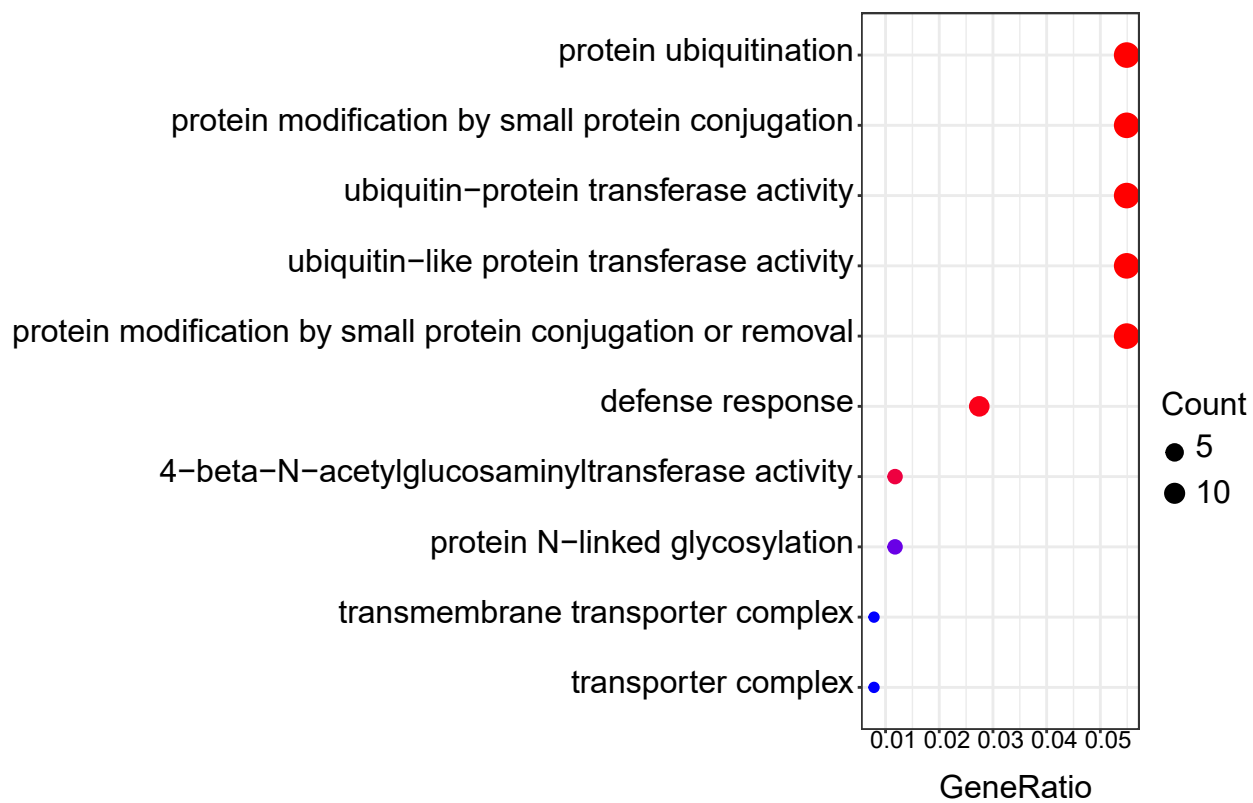

B

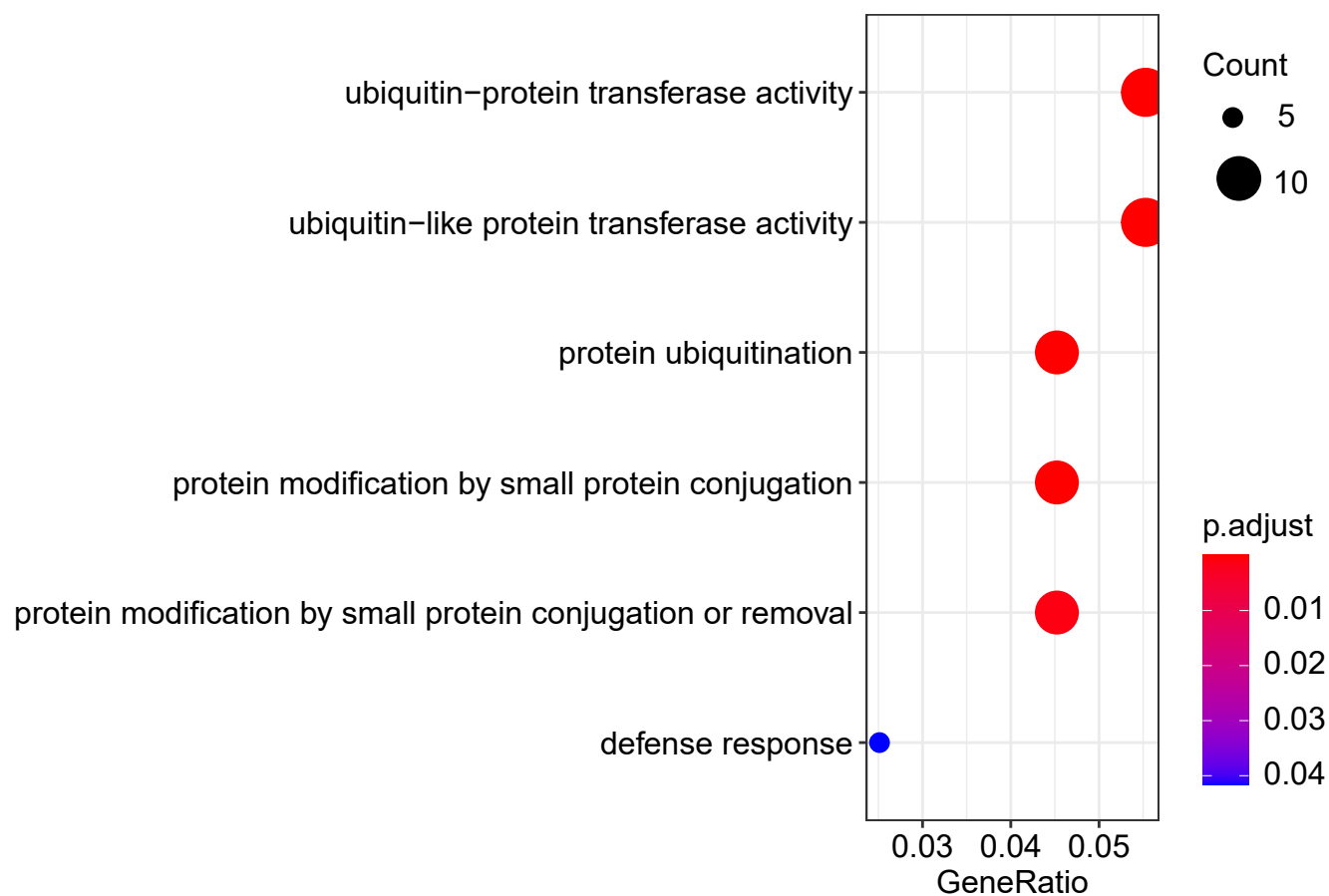

Supplement: Supplementary Figure 5 — GO categories of differentially expressed genes in all three comparisons. (A) Four hundred thirty-eight genes which are differentially expressed in leaves of both Cd10 vs. CK, Cd30 vs. CK, and Cd30 vs. Cd10. (B) Three hundred twenty-six genes which are differentially expressed in roots of both Cd10 vs. CK, Cd30 vs. CK, and Cd30 vs. Cd10. The x-axis indicates the ratio of genes vs. background gene numbers in each GO term. [file Image_5.PDF]
